# Comparison of surgical smoke between two approaches for endoscopic thyroidectomy and open thyroidectomy

**DOI:** 10.1186/s12893-022-01870-y

**Published:** 2022-12-08

**Authors:** Zhe Zhang, Gongsheng Jin, Xianfu Liu

**Affiliations:** The First Affiliated Hospital of Bengbu Medical College, 287 Changhuai Road, Bengbu, Anhui China

**Keywords:** Endoscopic surgery, Open surgery, Throidectomy, Particulate matter, Surgical smoke

## Abstract

**Background:**

Surgical smoke has been recognized as a potential health risk by an increasing number of researchers. Moreover, the counts of surgical smoke produced during different surgical approaches are different. This study aimed to measure and compare the particulate matter (PM) of surgical smoke generated during open thyroidectomy and two endoscopic approaches for thyroidectomy to provide guidance for safe clinical practices.

**Methods:**

Forty-eight patients with thyroid cancer admitted to our hospital from June 2020 to December 2021 and treated with different surgical approaches were enrolled in this study. The total and peak counts of PM, dynamic changes, and other characteristics of surgical smoke produced during surgery were recorded. PM was classified as PM_2.5_ (size ≤ 2.5 μm) and PM_10_ (size ≤ 10 μm).

**Results:**

In a single cut, both the peak and total counts of PM_2.5_ and PM_10_ of surgical smoke in the open thyroidectomy group (n = 15) were significantly higher than those in the breast approach endoscopic thyroidectomy with CO_2_ insufflation group (n = 15) and the gasless transaxillary endoscopic thyroidectomy group (n = 18) (p < 0.001). Moreover, the latter two groups showed no significant differences in the peak and total counts of PM_2.5_ and PM_10_ (*p* > 0.05).

**Conclusion:**

In thyroid surgery, more surgical smoke is produced during open thyroidectomy than during endoscopic thyroidectomy, while different endoscopic approaches showed no significant difference in surgical smoke production. Thus, endoscopic approaches outperform the open thyroidectomy approach with regard to surgical smoke production.

## Introduction

Surgical smoke mainly contains water vapor, and it is produced after membranolysis when energy-generating devices such as electric knives, ultrasonic knives, and lasers increase the intracellular temperature to over 100 °C [1]. Harmful substances produced by cell carbonization include benzene, cyanide, vinyl chloride, formaldehyde, methanol, and other carcinogens [2, 3]. As reported by the US Environmental Protection Agency (EPA), the inhalation of particulate matter (PM) with a diameter of 10 μm and below can induce several long-term chronic complications such as coronary artery disease, congestive heart failure, asthma, and chronic obstructive pulmonary disease [4]. A recent study showed that the inhalation of PM_2.5_ may damage mitochondria and enhance oxidative stress to influence the beating of cardiomyocytes [5]. Moreover, the inhalation of PM_10_ can trigger a Th1-shifting immune response in the lungs and possibly cause lung hypoxia, thereby influencing reproduction (fetal development) [6]. In addition to multiple compounds, cells, bacteria, and viral particles are also a part of surgical smoke [7], thus leading to the risk of infection. Several studies have reported the correlation of surgical smoke with human papillomavirus (HPV) infection [8–10]. Among the reported cases, two gynecologists developed tonsil infection of HPV16 type and oropharyngeal squamous cell carcinoma, possibly due to the transmission of surgical smoke [11]. In addition to HPV, hepatitis B virus (HBV) [12] and human immunodeficiency virus type 1 (HIV-1) [13] have also been detected in surgical smoke. However, because of the small number of corresponding cases reported, most medical workers do not have sufficient knowledge of the risks associated with surgical smoke. Indeed, more importance should be given to the infection risk of surgical smoke, especially in a period when global efforts are being made to control and prevent the novel coronavirus (COVID-19).

Research on surgical smoke has been conducted in various surgical fields, including gastrointestinal surgery, otolaryngology, urology surgery, and breast surgery [14–17]. However, limited research has been conducted on the characteristics of surgical smoke produced during thyroidectomy. The mainstream endoscopic approach in thyroid surgery is the gasless approach, where a cavity is formed by a suspended instrument. This is different from other surgical approaches in which gas is needed to maintain the cavity. Thus, it is important to investigate the characteristics of surgical smoke produced when different approaches are used in thyroid surgery. In the present study, the counts of PM in surgical smoke generated in a single cut in three thyroidectomy approaches and the dynamic changes of PM throughout the surgery were measured and recorded to determine the differences in surgical smoke produced during open thyroidectomy and endoscopic thyroidectomy approaches.

## Materials and methods

### Patients

Forty-eight patients with thyroid malignancies admitted to the First Affiliated Hospital of Bengbu Medical College from June 2020 to December 2021 were enrolled in this study. The research objective was explained to all the patients, and their written informed consent was obtained, which indicated their willingness to participate in the research and their explicit consent for publishing the research results. The following surgical approaches were used: open thyroidectomy (n = 15), breast approach endoscopic thyroidectomy with CO_2_ insufflation (n = 15), and gasless transaxillary endoscopic thyroidectomy (n = 18). The endoscopic approaches were selected according to patients’ condition and intentions. All the surgical procedures were completed by the same surgeon and his treatment team.

### Surgical methodology

#### Gasless transaxillary endoscopic thyroidectomy

The patient was placed in the supine position with the neck extended. The trachea was intubated through the mouth for anesthesia. After disinfecting and spreading the towel, an incision of 4–5 cm was made in the axillary, the subcutaneous space was separated to the surface of the pectoralis major muscle, a pull hook was placed toward the head of the clavicle, the flap was free at the marked range, and the surgical cavity was expanded, the strap muscle of the neck was dissociated to expose the thyroid gland. The pull hook is attached to the vacuum to quickly remove surgical smoke. After establishing the operation space, the position of the recurrent laryngeal nerve is first revealed, the inferior parathyroid gland is searched for, and preserved in situ as much as possible. Then, the recurrent laryngeal nerve is revealed along the surface of the esophagus, and the superior parathyroid gland is preserved in situ. The isthmus of thyroid gland and the anterior Delphian lymph nodes were removed after sequential dissection of the superior thyroid vessels and clearance of the central lymph nodes. The specimen is removed, the wound is flushed, and a negative pressure drainage tube is placed.

#### Breast approach endoscopic thyroidectomy

The patient was placed in the supine position with the neck extended. The trachea is anesthetized by tracheal intubation. An incision of approximately 1 cm is made in the medial areola, a 1.2 cm diameter trocar is placed, and a crossover tunnel is created subcutaneously toward the sternocleidomastoid muscle on both sides. The skin at the crossover was suspended with a silk thread, and then a 5 mm trocar was placed into the tunnel from both sides of the areola respectively, and after the subcutaneous freeing reached a certain extent, a pull hook was placed and connected to negative pressure suction to further complete the cavity building. From the linea alba cervicalis, the thyroid surgical capsule level is entered, and the pretracheal lymph node is cleared first, then the isthmus of the thyroid is disconnected, the anterior laryngeal lymph node is cleared upwards, and the superior thyroid artery is coagulated in a stepwise manner. The thyroid gland is removed from the bottom up, from the inside out avoiding the recurrent laryngeal nerve, and the parathyroid gland is preserved in situ as much as possible. In general, whole lobe, isthmus and central zone lymph node dissection is performed. The specimen is removed, the wound is irrigated and a drainage tube is placed from the areola area. In general, whole lobe, isthmus and central zone lymph node dissection is performed (en-block). The specimen is removed, the wound is irrigated, and a negative pressure drainage tube remained in the surgical space.

#### Open thyroidectomy

The patient was placed in the supine position with the neck extended. The trachea is anesthetized by tracheal intubation. The surgical incision was made in the superior sternal fossa at one transverse finger, reaching the medial edge of sternocleidomastoid muscle on both sides, and we injected an epinephrine-containing swelling (1:500,000 epinephrine) subcutaneously with normal saline to minimize bleeding from the incision. In general, whole lobe, isthmus and central zone lymph node dissection is performed (en-block). The pretracheal lymph node is cleared first, then the isthmus of the thyroid gland is disconnected, the ligament of Berry was excised, and then the cricothyroid space was entered upward, the superior thyroid artery was treated and the parathyroid gland was preserved, and finally the recurrent laryngeal nerve was revealed. Along the course of the recurrent laryngeal nerve and the surface of the esophagus, the thyroid lobe and the lymph nodes of the ipsilateral central region were excised in one piece, the specimen was removed, the wound was irrigated, and a drainage tube was placed and drained from the axilla.

#### Instruments and devices

The coagulation and cutting mode of the electrosurgical knives (HWGDJ-I-80, HUAWEITECH, CO, CHINA) was kept at 25–30 W, and the mode was blend cut. The operating frequency of the ultrasonic surgical devices (HARH36, HARMONIC, CO, USA) was adjusted to 55 kHz, and the vibration frequency of the knife head was adjusted at 50–100 μm, and the power was set at 3 grades.

A PM detector (CEM air quality monitor DT-9883M, CEMTECH, China), which is a laser PM counter for counting real-time numbers of PM_2.5_ and PM_10,_ was used to measure the PM counts of surgical smoke produced during the above-mentioned three surgical approaches. The opening of the PM detector was placed at 5 cm around the mouth and nose of the surgeon. Each cutting procedure lasted for over 1 s. After the cutting process began, the measurement started immediately and continued for at least 20 s.

### The environment in the operating rooms

All the operations were conducted in the same room, with an area of 36.5 m^2^, a volume of 113.15 m^3^, an ambient temperature of 20–24 °C, and an ambient humidity of 45–60%. The operating room was equipped with a high-efficiency PM air filter with an airflow of 3190–4270 m^3^/h and an air exchange rate of 36–41 times/h. The interval between the operations was over 1 h, and the PM number in the air of the operating room was counted before surgery to ensure that the background PM counts of each operation were consistent.

### Statistical analysis

SPSS Statistics 26.0 (IBM) was used for statistical analysis. The results are expressed as mean ± standard deviation. One-way analysis of variance (ANOVA) was used for the intra-group comparison of the PM measurement results of surgical smoke. Tamhane T2 test was used for inter-group comparison. All analyses were two-tailed, and statistically significant differences were considered at *p* < 0.05.

## Results

### Clinical characteristics

According to the general data of patients shown in Table 1, all of them received unilateral thyroidectomy and isthmus resection along with central lymph node dissection. The pathological result for all patients was papillary thyroid carcinoma, and none of the patients had any other thyroid diseases.

**Table 1:** Patient characteristics

Variables	Gasless transaxillary endoscopic thyroidectomy (n = 18)	Breast approach endoscopic thyroidectomy (n = 15)	Open thyroidectomy (n = 15)	p value
Age (mean, years)	30.5 ± 9.86	27.27 ± 5.79	44.6 ± 11.36	p < 0.001
Sex				
Male	2	1	6	
Female	16	14	9	
Height (mean, cm)	163.94 ± 3.84	162 ± 5.06	165.07 ± 6.03	p = 0.24
Weight (mean, kg)	61.5 ± 7.44	58.07 ± 6.73	64.53 ± 10.49	p = 0.12
Body mass index (mean, kg/m^2^)	23.03 ± 2.88	22 ± 1.95	23.62 ± 3.18	p = 0.27
Tumor size (mean, mm)	6.22 ± 3.04	6.07 ± 2.76	7.93 ± 4.8	p = 0.29
Operation time (mean, min)	154.83 ± 22.3	160.47 ± 17.55	99.87 ± 14.89	p < 0.001

### Comparison of PM counts among the three surgical approaches

**Fig. 1:**
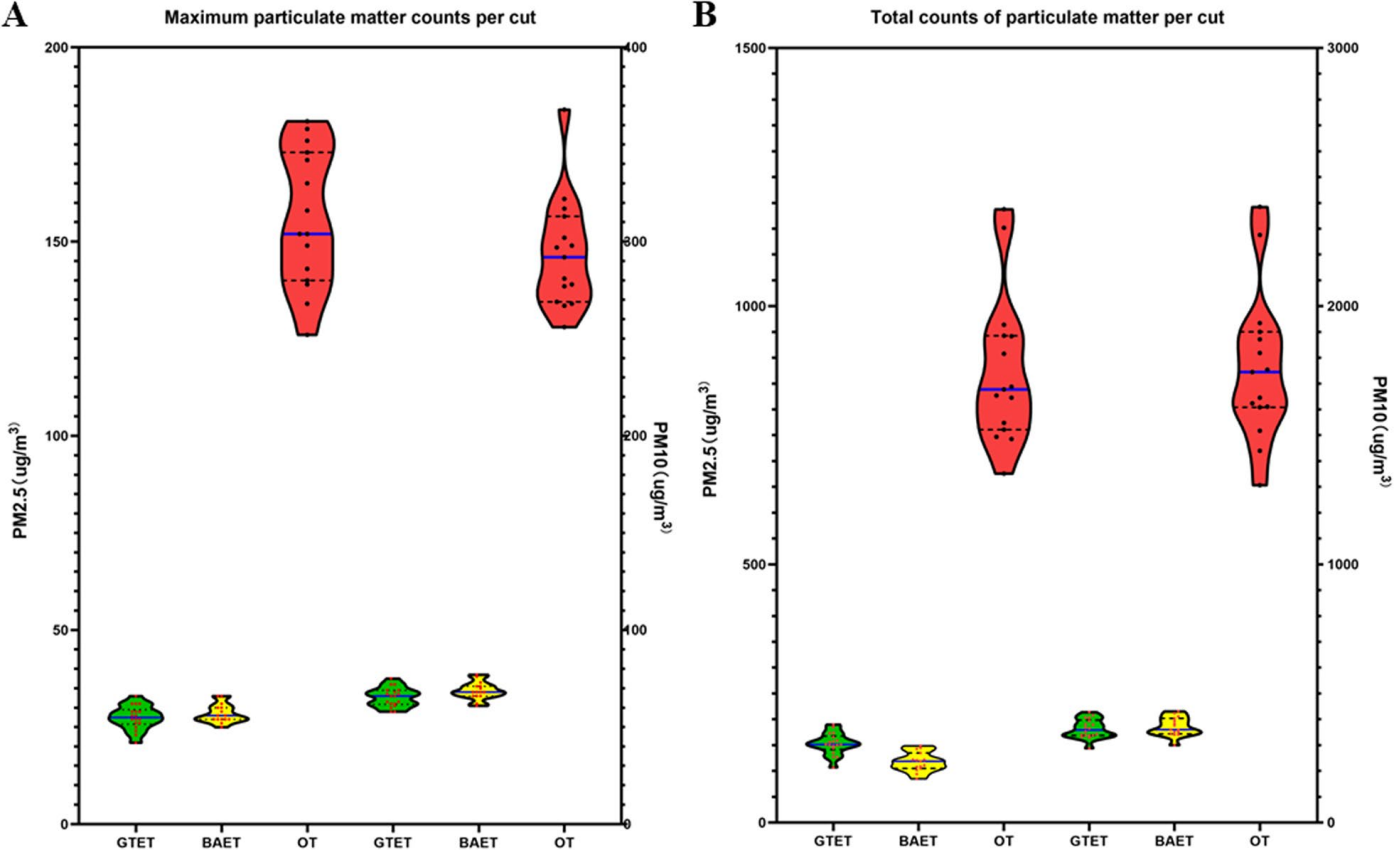
*GTET* Gasless transaxillary endoscopic thyroidectomy, *BAET* Breast approach endoscopic thyroidectomy, *OT* Open thyroidectomy

Figure 1 shows the peak and total counts of PM_2.5_ and PM_10_ of surgical smoke produced in a single cut. The peak counts of PM_2.5_ in surgical smoke in gasless transaxillary endoscopic thyroidectomy, breast approach endoscopic thyroidectomy with CO_2_ insufflation, and open thyroidectomy were 27.50 ± 3.07, 28.53 ± 2.41, and 155.9 ± 17.59 μg/m^3^, respectively, while those of PM_10_ were 65.44 ± 4.91, 68.73 ± 4.48, and 293.67 ± 4.48 μg/m^3^, respectively. The total counts of PM_2.5_ in a single cut of gasless transaxillary endoscopic thyroidectomy, breast approach endoscopic thyroidectomy with CO_2_ insufflation, and open thyroidectomy were 122.89 ± 20.83, 118 ± 18.83, and 875.40 ± 145.58 μg/m^3^, respectively, while those of PM_10_ were 363.44 ± 36.89, 371.40 ± 36.31, and 1762.80 ± 288.62 μg/m^3^, respectively. The peak and total counts of PM_2.5_ and PM_10_ in surgical smoke produced in a single cut were significantly higher in open thyroidectomy than in breast approach endoscopic thyroidectomy with CO_2_ insufflation and gasless transaxillary endoscopic thyroidectomy (*p* < 0.001), while the latter two approaches showed no significant differences in the peak and total counts (p > 0.05). However, the count of particles recorded throughout the operation was lower for open thyroidectomy than for two endoscopic approaches for thyroidectomy, but the number of particles generated per minute was higher than for two endoscopic approaches for thyroidectomy. These data are shown in Table 2.

**Table 2:** The analysis of the concentration number of particulate matter detected in three surgical approaches

	Particulate matter size	Maximum counts (one cut, means ± standard deviation)	Maximum counts (one cut, median)	Total counts (one cut, means ± standard deviation)	Total counts (one cut, median)	Total counts (whole procedure, means ± standard deviation)	Count per minute (means ± standard deviation)
Gasless transaxillary endoscopic thyroidectomy	2.5	27.50 ± 3.07	27.5	122.89 ± 20.83	121.5	1462.78 ± 102.55	9.55 ± 0.84
	10	65.44 ± 4.91	66	363.44 ± 36.89	359	2945.39 ± 238.45	19.21 ± 1.69
Breast approach endoscopic thyroidectomy	2.5	28.53 ± 2.41	28	118 ± 18.83	119	1464.53 ± 103.19	9.18 ± 0.63
	10	68.73 ± 4.48	68	371.40 ± 36.31	360	2971.60 ± 233.06	18.62 ± 1.41
Open thyroidectomy	2.5	155.9 ± 17.59	152	875.40 ± 145.58	839	1349.53 ± 161.19	13.64 ± 1.61
	10	293.67 ± 4.48	292	1762.80 ± 288.62	1745	2781.69 ± 343.26	28.10 ± 3.28

Unit: μg/m^3^

### Dynamic counts of PM in surgery

**Fig. 2:**
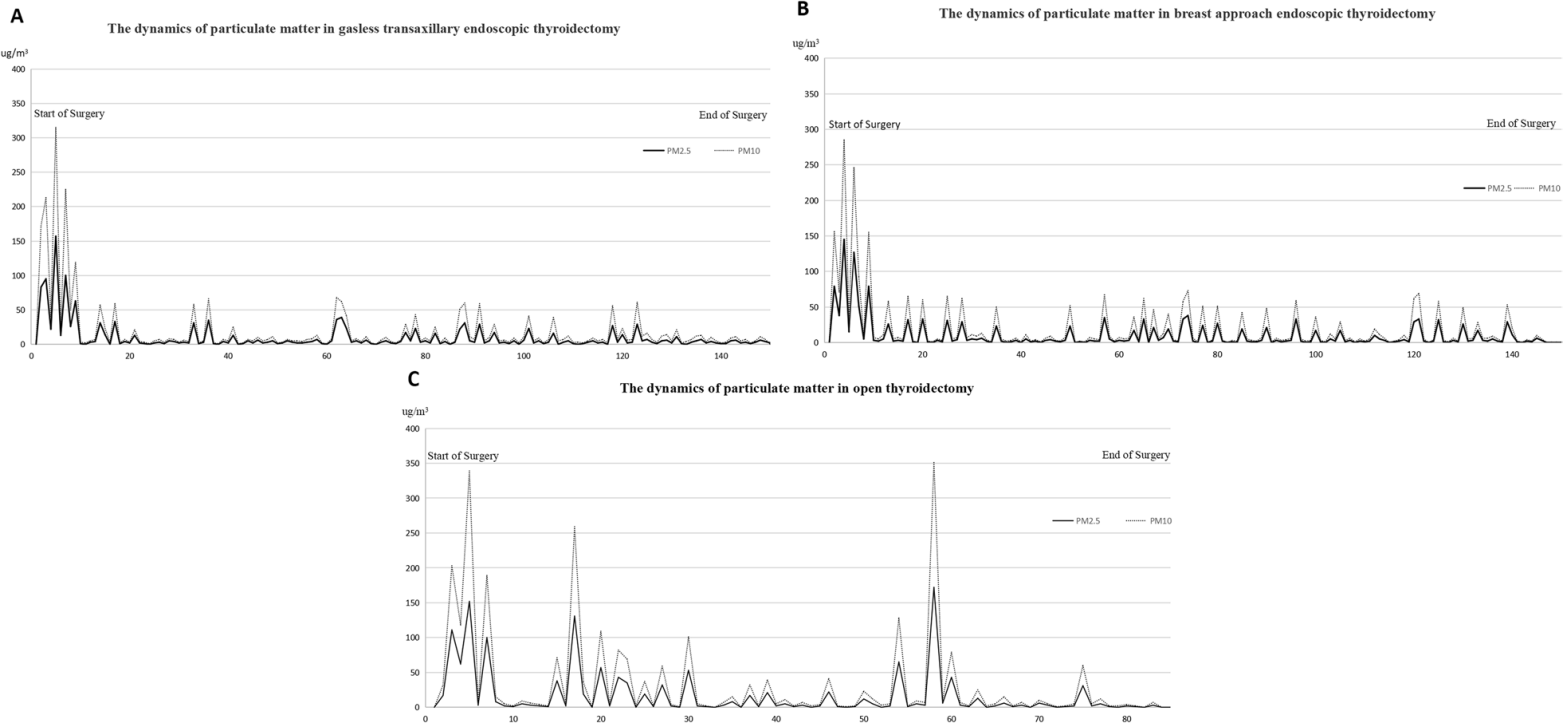
Dynamics of particulate matter counts in representative cases

The dynamic changes of PM_2.5_ and PM_10_ in the three groups are shown in Fig. 2. The included cases had similar indicators such as age, body mass index (BMI), and tumor size. As shown in the figure, the PM count and the occurrence frequency of high values in the surgical smoke of the open thyroidectomy group were significantly higher than those of the other two groups. In breast approach endoscopic thyroidectomy with CO_2_ insufflation and gasless transaxillary endoscopic thyroidectomy, only a small number of particles were released during intraluminal operations, while the increase in counts mainly occurred during extraluminal operations. As recorded, the concentration of PM_2.5_ reached the maximum of 172 μg/m^3^ during open thyroidectomy, where the air quality of the operating room was considered as “Very unhealthy” according to the air quality index (AQI) standard established by USEPA [18]. In contrast, the air quality in the operating room during the other two endoscopic approaches was mostly “Good” or “Moderate.”

## Discussion

In this study, the counts of PM contained in surgical smoke in a single cut and their dynamic changes throughout the entire surgical procedure of the three approaches of thyroidectomy were recorded and subjected to comparative analysis. The measured peak counts of PM contained in surgical smoke do not match with the results reported in other studies [16, 17]. This is because important blood vessels and nerves should be avoided in thyroidectomy, which require more delicate operations. Hence, it takes relatively short time to cut the tissue by an energy-generating device, thus reducing the production of cutting-generated smoke. Moreover, the concentration and particle size distribution of PM in surgical smoke also vary with the tissues treated. The number of PM produced in the liver is the largest, that produced in kidney tissue and skeletal muscle is moderate, and that produced in subcutaneous fat, lung tissue, bronchi, cerebral gray and white matter, and skin is evidently small [19]. The heterogeneity of the internal issue (such as the existence of connective tissues, vascular distribution, and hematoma) may also cause significant differences in the composition and volume of surgical smoke, even when the same specimen is used [19]. Hence, patients without other thyroid diseases were enrolled in this study, and their tumors were smaller than 20 mm in size. Given that the above-mentioned factors may lead to fibrosis, necrosis, lymphocytic infiltration, and other changes in thyroid pathology, the selection of such patients can reduce the influence of inter-tissue differences on the research results.

In gasless endoscopic thyroidectomy, a suction device is connected to the suspension retractor for the suction of surgical smoke, thereby affording better airflow in the operating cavity. In the breast approach endoscopic thyroidectomy with CO_2_ insufflation, the aggregated PM in surgical smoke at high concentrations is released via the trocar without filtering due to the presence of the stack effect [20], which may result in high-speed jets. Consequently, the level of emission of surgical smoke to the operating room is further increased. According to the PM counts of surgical smoke throughout the surgical process in this study, the two endoscopic approaches showed no significant difference in PM count. A possible reason for this is that the volume of smoke additionally ejected from the trocar is relatively small as the gas pressure required for cavity construction in endoscopic thyroidectomy is lower than that in laparoscopic surgery (only 8 mmHg).

Surgical smoke carries the risk of transmitting various viruses and bacteria. In the current COVID-19 pandemic situation, at the early epidemic stage, surgical societies are very cautious about endoscopic surgery because of concerns related to the infection risk posed by COVID-19-positive patients [21, 22]. As observed in the study of Cheruiyot et al*.*, the hypothesis that SARS-CoV-2 could be aerosolized and transmitted through surgical smoke is not proved by the existing evidence [23]. Nevertheless, the existence of this risk cannot be denied. Thus, any theoretical risk should be reduced by taking adequate measures such as choosing an appropriate surgical approach, correctly using personal protective equipment in the operating room, minimizing the power of energy-generating devices, avoiding long-term cutting procedures, frequently pumping to avoid smoke accumulation, and equipping smoke extraction systems in operating rooms with high-efficiency particulate air (HEPA) filters or ultra-low-penetration air (ULPA) filters [4, 15].

Various endoscopic and robotic approaches of thyroidectomy have been developed and continuously upgraded since 1997 to avoid the formation of neck scars. In a recent systematic review and meta-analysis, Lisa et al. compared the eight most commonly used approaches of endoscopic thyroidectomy with open thyroidectomy. Their results showed that endoscopic thyroidectomy does not prolong the length of hospital stay; moreover, the incidence of complications such as temporary/permanent recurrent laryngeal nerve injury and hypocalcemia after various endoscopic thyroidectomy approaches is lower or shows no significant difference from that after open thyroidectomy [24]. The results of the present study showed that endoscopic thyroidectomy is more preferable considering the risk of surgical smoke. We believe that the characteristics of surgical smoke measured in the two approaches of endoscopic thyroidectomy used in the present study are applicable to all minimally invasive approaches of thyroidectomy, as the tissue types and characteristics as well as operating cavities are similar.

The present study has some limitations. First, there was a sampling error in the cases included as endoscopic thyroidectomy is mainly selected by young women with a higher pursuit of beauty because of its primary feature of being “scar-free.” The diverse body compositions between males and females contribute to gender differences such as differences in fat distribution, brown adipose tissue, and muscle mass [25]. This may further increase the difference in surgical smoke between open and endoscopic thyroidectomy approaches [19]. In future research, data sampling should be improved to investigate whether this factor will influence surgical smoke production.

Second, the influence of energy-generating devices was not negligible. When such devices are used to heat the target tissue to the boiling point, cells rupture and produce surgical smoke. The setting of electrosurgical knives will influence the rate of tissue heating, thus influencing the properties of PM in surgical smoke and possibly the viability of pathogens [26]. Surgical smoke production by ultrasonic devices occurs through vaporization when the vibration of cells leads to relatively low temperature. The average size of PM produced by electrosurgical knives is smaller than 0.1 μm, while that of PM produced by ultrasonic surgical devices is 0.35–6.5 μm. Usually, smaller PM can travel longer distances and trigger more chemical effects related to surgical smoke, while larger PM may have a higher infection risk. Interestingly, although the size of PM generated by electrosurgical knives is smaller than that generated by ultrasonic devices, the latter devices produce more PM than the former ones [27]. This is probably one of the main reasons, besides the length of the procedure, that makes the total number of particles for endoscopic thyroidectomy greater than for open thyroidectomy, since the ultrasonic surgical devices is used much more frequently in endoscopic thyroidectomy than in open thyroidectomy. Hence, given the complex influences of energy-generating devices on surgical smoke, more in-depth investigation is necessary.

In this study, the dynamic changes of PM in surgical smoke and its counts in a single cut were investigated, which revealed significant differences in PM_2.5_ and PM_10_ counts between open and endoscopic thyroidectomy approaches. According to the results of the present study, even when a qualified air purification system is used, harmful PM produced during the use of energy-generating devices can still expose medical workers to a high-risk environment. Medical workers, however, often have to work in such a high-risk environment for a long time. Therefore, they should recognize such a potential health hazard, enhance their awareness of self-protection, and take adequate precautions against harmful surgical smoke. Currently, the main prevention methods are wearing surgical masks with strong filtering functions (such as N95 masks) and using activated carbon air filters combined with HEPA filters. The selection of appropriate surgical approaches is also another method of self-protection. The present study proves the difference in surgical smoke production among the several commonly used surgical approaches in thyroid surgery. Endoscopic approaches are evidently superior to the open surgical approach in terms of the degree of harm to medical workers. Though many influencing factors need to be further explored, the present research is still clinically relevant.

## Conclusion

In thyroidectomy, the open surgical approach produces more surgical smoke than endoscopic approaches, and different endoscopic approaches have no significant difference in surgical smoke production. Moreover, endoscopic approaches are superior to the open thyroidectomy approach in terms of surgical smoke. The risk of surgical smoke should not be underestimated, despite its smaller volume produced during thyroid surgery than during gastrointestinal surgery, gynecological surgery, breast surgery, and other surgeries. More importance should be given to this aspect, effective protective measures should be adopted, and more appropriate surgical approaches should be chosen.

## Data Availability

The data sets generated and analyzed during the current study are not publicly available due to restrictions on ethical approvals involving patient data and anonymity but can be obtained from the corresponding authors as reasonably required.
